# Renal Volume in ADPKD Patient Evaluation

**DOI:** 10.1155/2020/9286728

**Published:** 2020-02-25

**Authors:** M. Galliani, E. Vitaliano, S. Chicca, L. Calvaruso, L. Di Lullo, F. Iorio, M. E. Tosti, A. Paone

**Affiliations:** ^1^UOC Nefrologia, Dialisi e Litotrissia, Ospedale Sandro Pertini, Roma, Italy; ^2^UOC Nefrologia, Fondazione Policlinico Universitario A. Gemelli IRCCS, Roma, Italia, Università Cattolica del Sacro Cuore, Roma, Italy; ^3^UOC Nefrologia e Dialisi, Ospedale Delfino Prodi, Colleferro, Roma, Italy; ^4^UOC Diagnostica per Immagini, Ospedale Sandro Pertini, ASLRM2, Roma, Italy; ^5^National Center for Global Health, Istituto Superiore di Sanità, Roma, Italy

## Abstract

The clinical manifestations of ADPKD are related to the growth of renal cysts. Renal volume has been recognised as the biomarker that is able to identify those patients at risk of complications (hypertension and haematuria) and at risk of progression to End Stage Renal Disease (ESRD). Recently, several scores have been introduced to predict the evolution of ADPKD. The Mayo Clinic Group developed a classification based on renal volume as measured by CT or MRI and corrected for age and height (Ht-TKV); this allowed predicting the evolution of the disease, but it has not been fully validated so far. In addition, it is used to identify patients labelled as “fast progressors” and eligible for Tolvaptan therapy according to the European Renal Association-European Dialysis and Transplant Association (ERA-EDTA) recommendations. We studied 80 patients who underwent MRI and had been classified as ADPKD typical form (class 1A-1E). A significant correlation between renal volume, hypertension, and low GFR was found (*p* < 0.005). A progressive increase in disease severity has been found across the different Mayo classes; 41.2% were eligible for Tolvaptan therapy. The results demonstrate that the Mayo method is easy to perform and provides valid information in order to identify with rapidly progressing disease.

## 1. Introduction

Autosomal dominant polycystic kidney disease (ADPKD) is the most common hereditary kidney disease. About 10% of all dialysis patients (ESRD) are affected by this disease, but the optimal approach to disease monitoring and treatment remains uncertain [1].

Renal cysts are the hallmark of ADPKD; this anatomical alteration is associated with reduced urine concentration, renal stones, haematuria, pain, arterial hypertension, progressive GFR loss, and eventually ESRD [2]. Of note, these manifestations of ADPKD are associated with renal volume [3].

In the first decades of the disease, there is often no significant loss of renal function, probably due to hyperfiltration mechanisms [4, 5], which may cause a delay in the diagnosis of disease or an incorrect prognosis. Recently, renal imaging scores have been introduced to predict the evolution of ADPKD. The Mayo Clinic Group [6] developed a classification (Mayo Imaging Classification–MIC 1A–1E) based on renal volume as measured by CT or MRI and corrected for age and height (Ht TKV). Chapman [7], in an analysis from the CRISP study, showed that 30% of patients with Ht-TKV ≥600 ml/m go on to develop stage 3 chronic kidney disease (CKD) in an 8-year period.

The ellipsoid method by the Mayo Clinic Group was formally recommended by the ERA-EDTA to be used in daily clinical practice [8], and the evaluation of Total Kidney Volume (TKV) has become a valuable clinical tool for identifying patients who are eligible for treatment with Tolvaptan [9].

The objective of our study was to describe the application of the Mayo Clinic renal volume measurement in a series of outpatients affected by ADPKD.

## 2. Patients and Methods

We studied an incident series of 80 patients seen at the Nephrology Unit of Pertini Hospital (Table 1).

The Glomerular Filtration Rate (GFR) was calculated using the CKD-EPI formula. Hypertension was defined as sBP >140 mmHg and dBP >90 mmHg and/or the use of antihypertensive medications. MRI was performed with a GE 1.5 T device: weighted scans were performed in T1, T2, and DWI. The renal volume was calculated using the program available on the Mayo Clinic website [10].

The formula for volume calculation is based on the ellipsoid equation (*π*/6 × *L* × *W* × *D*). The parameters are obtained from the 4 larger measurements with respect to the AP, LL, SI, and transverse axes using the axial, coronal, and sagittal sequences. The volumes obtained are correlated with the patient's age and height (Ht-TKV) to obtain the reference class.

We excluded subjects with the atypical ADPKD form (class 2).

We identify patients eligible for Tolvaptan therapy according to the ERA-EDTA recommendations (*adult ADPKD patients aged <50 years with CKD stages 1–3a who have been demonstrated to have a rapidly progressing disease*) [9].

## 3. Statistical Methods

The characteristics of the study population were described by median plus Interquartile Ranges (IQR) and compared between groups by the nonparametric Kruskal–Wallis rank test when represented by continuous variables. Discrete characteristics were described using proportions and compared between groups by *χ*^2^ (of Fisher's exact test, as necessary) or *χ*^2^ for trend tests. In Tables 2 and 3, the *p* value indicates the level of significativity of the association between hypertension (%), GFR <60 ml/min (%), and haematuria (%) with age (classes). In Table 4, the *p* value indicates the level of significativity of the association between *PKD1*/tot. genotyped (%), *PKD1* truncating/nontruncating(%), hypertension (%), GFR<60 ml/min (%), and haematuria (%) with MIC. A *p* value < 0.05 was considered as statistically significant. All statistical analyses were performed by STATA/SE 13.1.

## 4. Results

In this population, 78.8% are hypertensive, 55% show a reduction in renal function, 32.5% report episodes of haematuria, and 36.2% show a family history of ESRD <55 years of age. Among the 28 patients where genetic studies could be performed, 22 (78.6%) had a *PKD1* gene mutation, 12 (54.5%) had a truncating mutation and correlated to MIC D-E. The median Ht-TKV was 1220 ml/m (range 136.9–5962).

Table 2 shows the prevalence of arterial hypertension, haematuria, and GFR <60 ml/min/1.73 m^2^ across age strata. Hypertension (*p*=0.020) and low GFR (*p*=0.037) increase in parallel with age, while haematuria is evenly distributed across the various age strata.

As shown in Table 3, the prevalence of hypertension (*p* < 0.001) and GFR <60 ml/min/1.73 m^2^ (*p* < 0.001) increases progressively across kidney volume strata, while haematuria is more frequent in patients with Ht-TKV >701 ml/m.

Table 4 shows the clinical features and complications distributed according to the Mayo Imaging Classification. Low GFR (*p* < 0.001) is increasingly more prevalent across MIC 1A–1E, denoting increasing disease severity. Likewise, a similar tendency seems to exist for *PKD1* genotype, hypertension, and haematuria, but none of these variables reaches statistical significance (*p*=0.128, 0.184 and 0.245, respectively). Overall, 33 patients (41.2%) were eligible for Tolvaptan therapy.

## 5. Discussion

In this study, we confirm that alterations in renal volume (expressed as Ht-TKV) are associated with the main clinical manifestations of ADPKD (see Table 3). Grantham [3] reported TKV values > 700 ml as the mean TKV associated with complications among ADPKD patients. Furthermore, it is well demonstrated that GFR loss over time is dictated by total kidney volume [11], being the most considerable progression rate to ESRD rate (over 5 ml/min/year) observed as soon as the renal volume overcomes 1500 ml in patients older than 30 years.

In our population, about 3/4 of the population showed large kidney volumes (MIC 1C–1E) while the proportion of patients with a GFR <60 ml/min/1.73 m^2^ was 55%. This observation once again confirms that the measurement of total kidney volume provides important information regarding GFR for the proper clinical framing of ADPKD [12].

In our study, 70 patients (over 87%) were classified in the MIC 1C–1E which represent those with the highest progression rate (estimated loss of GFR from −2.63 to −4.78 ml/min/1.73 m^2^ year in males and from −2.43 to −4.58 ml/min/1.73 m^2^ year in females). Unlike other disease-predictive scores [13], the method proposed by the Mayo Clinic is exclusively based on the measurement of kidney volume. Among the patients who underwent TKV measurement by means of MRI, the authors identified subjects with the typical form (Class 1) of ADPKD (590 pts from Mayo and 173 from the CRISP study) and defined 5 subclasses on the basis of the annual growth of volume (1A–1E). From analysis of the GFR slope during the follow-up, each class was labelled with an increasing estimated risk for annual GFR loss (from −0.23 to −4.78 ml/min/1.73m^2^ in males, and from 0.03 to −4.58 ml/min/1.73m^2^ in females). It is worth noting that in this population, the GFR was calculated using the CKD-EPI formula. In addition, the frequency of subjects with a *PKD1* mutation increased in cases of greater disease severity (as in our population) [6]. A recent Swiss study of 214 patients with a mean age of 34 years and a typical ADPKD form for a follow-up of approximately 10 years confirmed the predictive model of the Mayo Clinic [8].

According to ERA-EDTA recommendations, the definition of “evidence of rapid progression disease” can be established by instrumental, clinical, or genetic criteria; at the same time, anamnestic data on eGFR, TKV changes, and/or genotype information are not always available. MIC can evaluate disease's progression using a single TKV value, and it could be easier to apply in clinical practice.

In the present study, 33 of 70 patients (47.1%) belonging to the MIC 1C–1E were eligible for Tolvaptan therapy.

Whether the method proposed by the Mayo Clinic based on the calculation of the ellipsoid is adequate for the correct measurement of renal volume is still debated [14].

Recently, semiautomatic or fully automated techniques for renal volume evaluation have been developed: these show good correlation with manual planimetry techniques being recommended for clinical practice [15].

The Mayo Clinic method even now appears to be the simplest and most reliable instrument for quantifying renal volume. Despite a recent survey carried out in Belgium having shown that magnetic resonance in these patients is rarely used, except in selected cases [16], we emphasize that these technique results the one of choice because of the safety, no ionizing radiation, panoramic approach, and elegibility for follow-up comparison.

A study based on the data of an American insurance company documented that the health costs of patients with ADPKD increase according to the different stages of CKD, becoming particularly high in the advanced stages and in ESRD (about 5000 dollars/year more for each ml/min reduction of GFR in subjects with GFR <30 ml/min) [17]. Whether increasingly sensitive and effective renal imaging studies improve the clinical management and reduce the health costs of ADPKD is an issue of primary importance. The challenge we have to face today is all about finding a trade-off between the need for sensitive and effective renal imaging tools, in order to improve clinical management and the effort to contain the ADPKD health costs.

## 6. Conclusions

Although performed on a small number of patients, this study confirms the simplicity and the potential usefulness of the measurement of renal volume by the Mayo Clinic method. This method provides essential information to nephrologists, being highly effective at identifying patients eligible for Tolvaptan therapy.

## Figures and Tables

**Table 1:** Clinical signs at the time of MR.

Age (median + IQR)	46.3 years of age (36.8–52.5)
M/F	37/43
Creatinine (median + IQR)	1.4 mg/dl (1.0–2.3)
eGFR (CKD-EPI) (median + IQR)	58 ml/min/1.73 m^2^ (30.2–82.8)
Ht-TKV (median + IQR)	1207 ml/m (704–2116)
Hypertensive patients (%)	63 (78.8%)
Patients with haematuria (%)	26 (32.5%)
Patients with GFR <60 ml/min/1.73 m^2^ (%)	44 (55.0%)
Family history for ESRD <55 years (%)	29 (36.2%)
*PKD1*/tot. genotyped (%)	22/28 (78.6%)
*PKD1* truncating/nontruncating (%)	12/22 (54.5%)

**Table 2:** Hypertension, low GFR, and haematuria across age strata.

Age	Hypertension (%)	GFR <60 ml/min/1.73 m^2^ (%)	Haematuria (%)
≤35 (17)	52.9	41.2	35.3
36–45 (21)	76.2	38.1	42.9
46–55 (30)	90.0	63.3	26.7
>55 (12)	91.7	83.3	25.0
*p* value	0.020	0.037	0.618

**Table 3:** Hypertension, low GFR, and haematuria across Ht-TKV strata.

Ht-TKV ml/m	Hypertension (%)	GFR <60 ml/min/1.73 m^2^ (%)	Haematuria (%)
≤700	50.0	10.0	10.0
701–1500	74.1	44.4	48.1
>1500	100.0	90.9	33.3
*p* value	≤0.001	<0.001	0.153

**Table 4:** Genotypes, hypertension, low GFR, and haematuria across MIC.

MIC	*PKD1*/tot. genotyped	*PKD1* truncating/nontruncating	Hypertension (%)	GFR <60 ml/min/1.73 m^2^ (%)	Haematuria (%)
1A/4	2/3 66.7%	0/2 0%	(2) 50%	(0) 0%	(0) 0%
1B/6	2/3 66.7%	0/2 0%	(5) 71.4%	(2) 28.6%	(1) 14.3%
1C/28	6/10 60%	0/6 0%	(19) 70.4%	(13) 48.2%	(7) 25.9%
1D/21	6/6 100%	6/6 100%	(18) 85.7%	(12) 57.1%	(8) 38.1%
1E/21	6/6 100%	6/6 100%	(19) 90.5%	(17) 81.0%	(10) 47.6%
*p* value	0.128	<0.001	0.184	<0.001	0.245

## Data Availability

The research data can be provided by the authors.
